# The levels and trends of cancer incidence in the elderly population at national and sub‐national scales in Iran from 1990 to 2016

**DOI:** 10.1002/cnr2.1937

**Published:** 2023-12-04

**Authors:** Zahra Shokri Varniab, Sahar Saeedi Moghaddam, Ashkan Pourabhari Langroudi, Sina Azadnajafabad, Seyede Salehe Mortazavi, Ali Sheidaei, Kimiya Gohari, Yosef Farzi, Zeinab Shirzad Moghaddam, Hanye Sohrabi, Mohsen Shati

**Affiliations:** ^1^ Non‐Communicable Diseases Research Center, Endocrinology and Metabolism Population Sciences Institute Tehran University of Medical Sciences Tehran Iran; ^2^ Endocrinology and Metabolism Research Center, Endocrinology and Metabolism Clinical Sciences Institute Tehran University of Medical Sciences Tehran Iran; ^3^ Geriatric Mental Health Research Center, School of Behavioral Sciences and Mental Health (Tehran Institute of Psychiatry) Iran University of Medical Sciences Tehran Iran; ^4^ Department of Epidemiology and Biostatistics, School of Public Health Tehran University of Medical Sciences Tehran Iran; ^5^ Department of Biostatistics, Faculty of Medical Sciences Tarbiat Modares University Tehran Iran; ^6^ Mental Health Research Center, Department of Epidemiology, Psychosocial Health Research Institute Iran University of Medical Sciences Tehran Iran

**Keywords:** elderly, epidemiology, geriatrics, incidence, neoplasms

## Abstract

**Background:**

Cancer is most commonly associated with aging. It is necessary to gain a better understanding of cancer's trend and distribution among elderlies and provide comprehensive cancer care for this population.

**Aims:**

The aim of the current study was to show the trends in cancer incidence focusing on the population aged 60+ from 1990 to 2016 in Iran.

**Material and Results:**

We used the dataset of the Iran Cancer Registry to estimate cancer incidences by sex, age, province, and year. In order to account for incomplete data we used a two‐stage spatiotemporal model along with random intercept mixed effect models. We calculated annual age‐standardized incidence rates (ASIRs) for age groups 60+ and 5‐interval age groups. There was an increasing trend of 25.3% to 936.9% (95% uncertainty interval: 769.6–1141.8) in ASIR in the elderly in 2016. ASIR of all cancers were 889.7 (731.3–1083.6) in women and 988.1 (811.1–1205) in men in 2016, per 100 000 respectively, which had an increasing trend comparing 1990. Skin, breast, and stomach cancers in women and prostate, skin, and stomach cancers in men were the most common types in 2016. All the most incident cancer subtypes underwent an increasing trend in both sexes, except for the bladder, esophageal, and skin cancers which almost had a similar level in 1990 and 2016. Most provinces had an increasing trend in ASIR in all cancers combined from 1990 to 2016 except Zanjan with a decreasing trend.

**Conclusion:**

Regarding the persistent increasing trend of most elderly cancers' incidence, this is crucial for policymakers to establish preventive plans, determine proper resource allocation, and develop specific treatments for elderly cancer patients.

## 1 INTRODUCTION

Cancer in the elderly population (older adults aged 60+) has increased worldwide due to the dramatic rise in life expectancy and aging of the population which is estimated over the next two decades double in globally.^1^, ^2^, ^3^, ^4^, ^5^ As a result of population aging, the cancer incidence in the elderly population is rising, about 11‐fold compared to younger adults,^6^ and results in cancer in older individuals causing a significant health concern. According to a census conducted in Iran, the number of older people has increased from 7.3% in 2006 to 8.65% in 2016, and it is expected to reach 21.7% by 2050.^7^ The rapidly growing elderly population and rising cancer incidence as its consequence will increase the demand for cancer care in this population, which will have a substantial impact on healthcare resource allocation.

Other than aging, geographical differences can affect cancer incidence. Moreover, cancer‐specific reports are helpful in improving healthcare management and in understanding the burden of cancer among specific age groups.^8^

As, Iran is witnessing a demographic shift and aging, also geographical investigation and gender differences can provide policymakers with new opportunities to understand cancer status and trends, environmental or geographical risk factors, effective prevention, targeted interventions. According to our knowledge, this is the first study to examine cancer levels and trends at the national and subnational levels in Iran. According to a previous study, the incidence of cancer among the elderly was demonstrated only in Tehran.^9^ The aim of the present population‐based study in Iran was to give an overview of the trends in cancer incidence in Iran from 1990 to 2016 in the elderly population older than 60 years at national and sub‐national scales.

## 2 METHODS

The methods used in this study are briefly described here, more details were brought in other papers.^10^, ^11^, ^12^, ^13^

### 2.1 Patient and public involvement

None.

### 2.2 Data source

This paper is the cancer subset of a comprehensive study called NASBOD (National and Sub‐national Burden of Diseases, Injuries, and Risk Factors), which aimed to estimate the burden of diseases and injuries by location (national and 31 provinces), sex, age groups, burden measures (incidence, prevalence, death, DALYs, YLLs, and YLDs) from 1990 to 2016.^14^ We used the Iran National Cancer Registry (INCR) databases of the Cancer Registration System (CRS) (from 2000 to 2010, excluding 2006), which were made available by the Ministry of Health and Medical Education of Iran. The main sources for data collection included pathology, radiotherapy, chemotherapy, and surgical centers, hospitals. The main variables in this study included age, sex, and place of residence (at the district level), codes of diagnosed cancers, year of first cancer diagnosis, first names, surnames, parents' names, postal address, and other identifying characteristics. Cancer types were classified according to the International Classification of Diseases for Oncology (ICD‐O), and then mapped to 18 major groups and 70 subtypes of cancer. The diagnosis of cancer was recorded by the attending physicians who initially examined the patients. The Social Security Organization (SSO) covers almost 40% of the population in Iran and has a complete registry of the financial insurance services of the registered cancer patients. Using the SSO cancer registry data, the incompleteness rate of the INCR was calculated for each year; given the extremely high cost of cancer drugs and hospitalization, the insurance organizations have almost 100% coverage for the registered cancer patients. Based on the SSO registry, we observed an incomplete rate of 78% in 2000 and a complete rate of 25% in 2010 for all diseases at all ages.^10^ Iran's provincial divisions have changed over the past 27 years; these changes were secondary to the transfer of some cities from their original province to another province and the division of some larger provinces into smaller ones. To correct for this misalignment, we used information from 2011, when the country was divided into 31 provinces. Wealth index, years of education, and urbanization rate were extracted from the Household Income and Expenditure Surveys and the population and housing censuses of the Statistical Center of Iran. All‐cause incidence rates were modeled in two stages. First, we used a random intercept mixed effects model, and in the next step, we remodeled the residuals from this model using an age‐spatio‐temporal model. In the first phase, we included years of schooling, wealth index, and urbanization as fixed effect covariates and province as a random effect. It is assumed that the residuals extracted from this model have some variation that cannot be captured in this model. The age‐spatio‐temporal model is used to account for variances and correlations among age groups, time, and provinces. Three weight matrices were developed through it, adjusted years, age groups, and provinces get more weights than others. In the case of location, weights were applied considering neighboring provinces, and in the case of age groups, the weight matrix was an applied function of age difference between age groups combined with a smoothing hyperparameter. Cancer subtype incidence data were modeled by multinomial logistic regression. We further applied a Bayesian model for multinomial distribution and overdispersion in this logistic regression. The 95% uncertainty intervals (UIs) were estimated by simulating the aggregated data based on variations in the individual data.

### 2.3 Data preparation

INCR contains two forms of duplicate data; the first is caused by a patient visiting multiple physicians to cross‐validate the results of a positive pathological test; whereas the second type of duplicate entry occurs when a patient has several entries over a period of time.

Except for patients with various cancers recorded in different years, we removed all duplicate data. We used 95% UIs to present the incidence. For reporting data in Age‐standardized rates, we used the direct method of standardization Based on the Iranian elderly population in 2016. Using decomposition analysis, we examined the impact of population growth, population aging, and age‐specific incidence rates on the absolute change in new cancer cases between 1990 and 2016. Two sets of hypothetical data were modeled: (1) The age and sex composition as well as the age‐specific rates from 1990 were applied to the overall population of 2016; (2) The age‐specific rates from 1990 were applied to the age and sex composition as well as the population in 2016. Population aging has been attributed to the difference between the second and first hypothetical data. The differences between the incidence rates of new cases in 2016 and hypothetical data were explained by the changes in the incidence rates of specific age groups.^15^ We calculated the max/min ratio in 1990 and 2019 for ASIR by gender to describe the disparities in 31 provinces. In cases where there were no considerable differences among them, we reported the maximum and minimum ASIR among all 31 provinces. A detailed discussion of the methodology of these procedures has been published previously.^10^, ^11^, ^12^, ^13^, ^16^, ^17^ We used data of all age populations to report the incidence of cancer in the elderly compared to all ages.

### 2.4 Study variables

The main variables included in this study include province, age at diagnosis, sex, and type of cancer. There are 18 major groups and 70 subgroups of cancers included in our study as seen in (Table S1) based on the ICD‐O, for 5 age groups (60–64, 65–69, 70–74, 75–79, 80+) by sex. Numbers of new cases among adults aged 60 years and older and truncated age‐standardized incidence rates (ASIRs) per 100 000 for all cancer sites combined and the cancers accounting for more than 50% of all cancers among men and women, were reported at national and provincial levels from 1990 to 2016. As a standard population, Iran's population in 2016 was used.

### 2.5 Statistical analysis

R programming platform (version 3.0.2) and STATA software (version 11.0) were used to conduct the statistical analysis. In the initial analysis, missing data were derived from the available individual data. The missing data were calculated using the Amelia package in the R programming language.^18^ The text mining algorithm of Python software (version 2.7.4) was used to search for duplicated data.

## 3 RESULTS

### 3.1 All‐cause cancer

**FIGURE 1:**
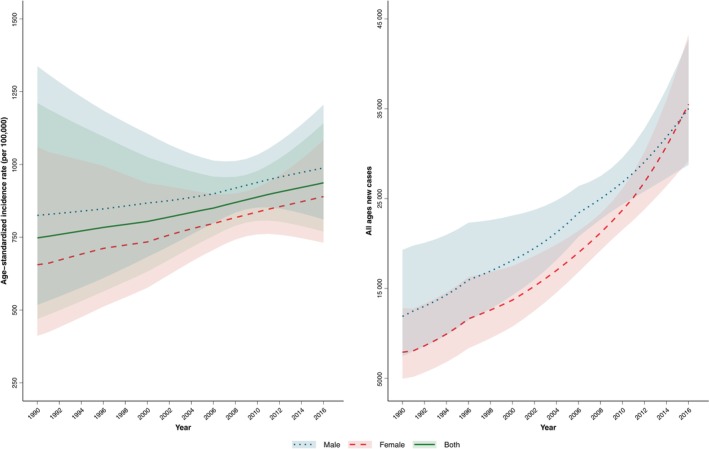
The trend of the age‐standardized incidence rate and the number of all cancer combined per 100 000 population during 1990–2016 in elderly men, women, and both in Iran.

**FIGURE 2:**
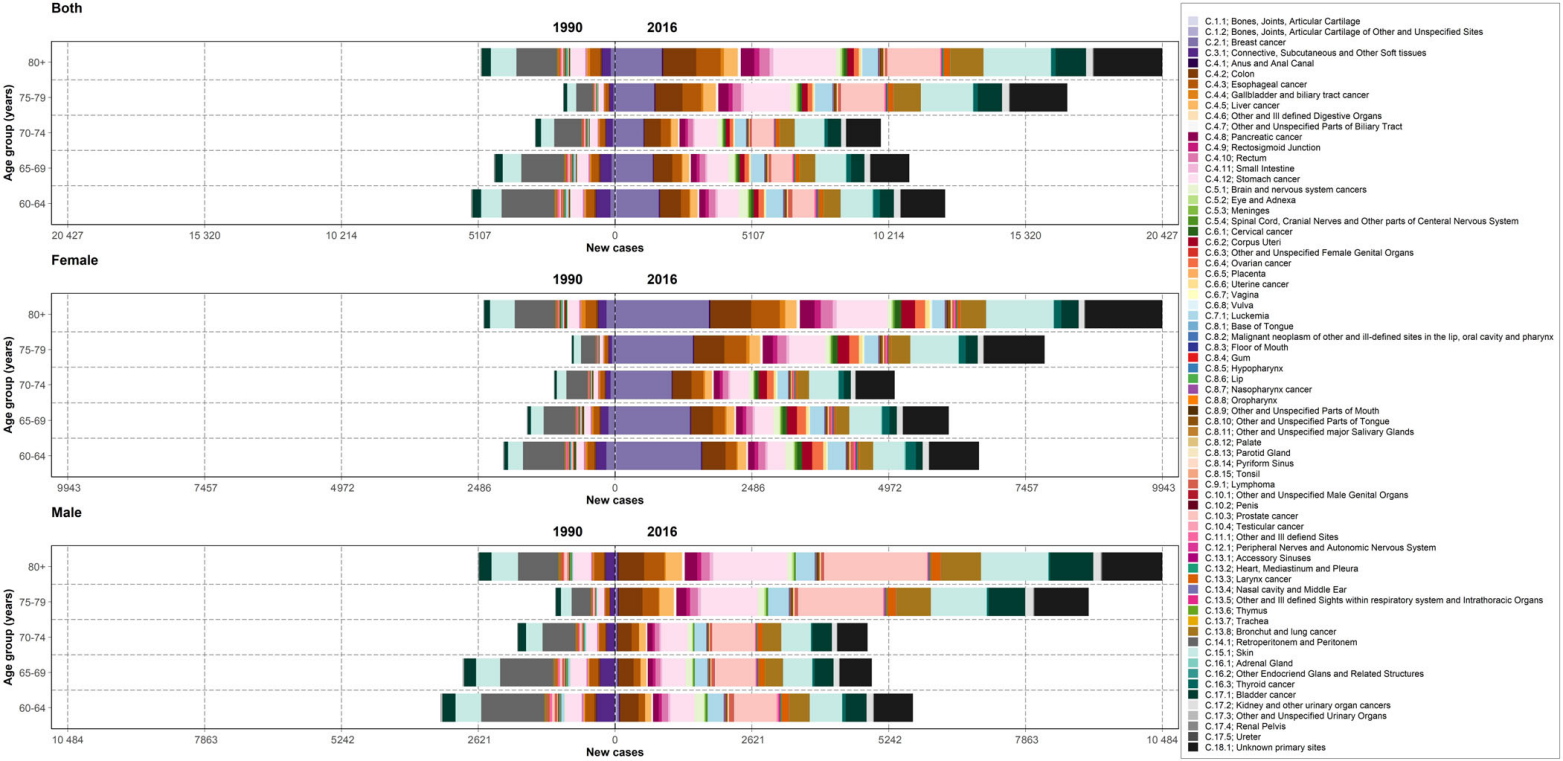
The incidence number of all 70 subgroups of cancers by age and sex in the elderly population between 1990 and 2016 in Iran.

Overall, in 2016, 70 520 (95% UI: 57929–85 943) new cancer cases were identified in the elderly in Iran which has undergone an increasing trend from 19 805 (12420–32 100) in 1990. (Figures 1 and 2) (Table 1) ASIR in the elderly was 936.9 (769.6–1141.8) per 100 000 at the national level in 2016 which had an increasing trend compared to 1990, 747.8 (469.2–1211.0). (Table 2) Based on the decomposition analysis, out of the 256.1% change in the total new cases, there was a 150.1% change attributable to population growth, 34.1% change in age structure, and 71.9% change in incidence rate. The elderly aged 80+ years old had the worst crude incidence rate (CIR) in 2016 with 1973.6 (1619.3–2408.2) per 100 000 and the lowest was among the elderly with 60–64 years with 504.1 (414.2–614.1). On the other hand, with increasing age, the CIR increased dramatically. All age groups and all years showed a persistent male predominance in CIR. Based on the CIR, cancer was almost more than 12‐fold more frequent among the elderly compared with the population aged less than 60, 1008.9 (828.2–1230.3) and 75.9 (62.3–92.5), respectively (Table 1).

**TABLE 1:** Number and crude incidence rate (CIR) per 100 000 of all cancers by age group, sex, and year at the national level between 1990 and 2016 in Iran.

Age	Sex	1990		2016	
		Number	Rate (per 100 000)	Number	Rate (per 100 000)
All ages	Both	44 255 (27 781–71 629)	81.8 (51.4–132.5)	126 982 (104 304–154 770)	155 (127.3–188.9)
	Female	18 176 (11 414–29 404)	69.2 (43.4–111.9)	62 882 (51 668–76 618)	153.8 (126.4–187.4)
	Male	26 079 (16 367–42 225)	93.8 (58.9–151.9)	64 100 (52 637–78 151)	156.2 (128.3–190.5)
<60 years	Both	24 449 (15 362–39 528)	47.9 (30.1–77.4)	56 462 (46 375–68 826)	75.9 (62.3–92.5)
	Female	10 268 (6454–16 597)	41.2 (25.9–66.6)	27 378 (22 484–33 377)	74.3 (61.1–90.6)
	Male	14 181 (8908–22 932)	54.2 (34.1–87.7)	29 084 (23 891–35 449)	77.4 (63.6–94.4)
≥60 years	Both	19 805 (12 420–32 100)	658.1 (412.7–1066.6)	70 520 (57 929–85 943)	936.9 (769.6–1141.8)
	Female	7908 (4960–12 807)	585.6 (367.3–948.3)	35 504 (29 183–43 241)	875.3 (719.5–1066.1)
	Male	11 898 (7460–19 294)	717.1 (449.6–1162.9)	35 016 (28 746–42 702)	1008.9 (828.2–1230.3)
60–64 years	Both	5368 (3371–8688)	421.5 (264.7–682.2)	12 317 (10 122–15 006)	504.1 (414.2–614.1)
	Female	2028 (1274–3282)	356.9 (224.2–577.5)	6612 (5436–8052)	490.9 (403.6–597.8)
	Male	3340 (2097–5406)	473.6 (297.3–766.6)	5705 (4686–6953)	520.4 (427.4–634.2)
65–69 years	Both	4516 (2835–7318)	559.2 (351.1–906.3)	10 981 (9030–13 368)	698.6 (574.5–850.4)
	Female	1597 (1002–2587)	463 (290.5–750)	6063 (4987–7380)	684.4 (562.9–833.1)
	Male	2918 (1833–4730)	631.1 (396.3–1022.9)	4917 (4043–5988)	716.8 (589.4–872.8)
70–74 years	Both	2983 (1874–4822)	685.8 (431–1108.5)	9918 (8137–12 102)	830.1 (681.1–1012.9)
	Female	1108 (696–1788)	574.9 (361.2–928.3)	5081 (4173–6194)	791.3 (649.9–964.6)
	Male	1876 (1179–3033)	774 (486.4–1251.9)	4837 (3964–5908)	875.2 (717.3–1069)
75–79 years	Both	1930 (1212–3124)	1022.1 (641.5–1653.8)	16 877 (13 881–20 544)	1316.6 (1082.9–1602.7)
	Female	788 (495–1272)	911.2 (572.1–1471.6)	7805 (6422–9496)	1219.1 (1003.1–1483.3)
	Male	1143 (717–1851)	1115.8 (700.2–1807.6)	9073 (7459–11 048)	1413.9 (1162.5–1721.8)
≥80 years	Both	5008 (3128–8149)	1643.1 (1026.3–2673.7)	20 426 (16 759–24 924)	1973.6 (1619.3–2408.2)
	Female	2387 (1493–3877)	1509.7 (944.6–2452.2)	9942 (8166–12 119)	1838.1 (1509.7–2240.5)
	Male	2621 (1635–4272)	1786.8 (1114.3–2912.4)	10 484 (8593–12 805)	2122 (1739.4–2591.8)

**TABLE 2:** The age‐standardized incidence rate of 18 main groups and 70 subgroups of cancers in both sexes combined in the elderly population between 1990 and 2016 in Iran.

Cause code	Cause name	Year			
		1990	2000	2010	2016
Total all cancers	**All cancers**	**747.8 (469.2–1211)**	**804.2 (632.3–1025.5)**	**888 (806.4–977.9)**	**936.9 (769.6–1141.8)**
C.1	**Bones, joints, and articular cartilage**	**0.8 (0–1.6)**	**1.7 (0.8–2.5)**	**2.1 (1.9–2.4)**	**2.2 (1.3–3)**
C.1.1	Bones, joints, and articular cartilage	0.2 (0–0.5)	0.5 (0.2–0.8)	0.6 (0.5–0.8)	0.7 (0.4–1)
C.1.2	Bones, joints, and articular cartilage of other and unspecified sites	0.6 (0–1.2)	1.2 (0.6–1.8)	1.5 (1.2–1.8)	1.5 (0.9–2.2)
C.2	**Breast cancer**	**22.2 (15.5–28.9)**	**53 (45.5–60.6)**	**80.3 (77.6–83)**	**94.7 (84.5–104.8)**
C.3	**Connective, subcutaneous, and other soft tissues**	**59.7 (15.2–104.2)**	**22 (12.6–31.3)**	**4.4 (4–4.9)**	**1.5 (1–2)**
C.4	**Digestive organs**	**156.4 (135.3–178.2)**	**272.8 (253.1–292.9)**	**290.8 (285–296.7)**	**266.7 (249.7–284)**
C.4.1	Anus and anal canal	1.7 (0.7–2.7)	2.4 (1.5–3.3)	1.8 (1.2–2.5)	1.3 (0.7–1.8)
C.4.2	Colon cancer	9.5 (7.7–11.4)	30.7 (27.3–34.2)	52.4 (48.8–56.1)	57.7 (51.7–63.8)
C.4.3	Esophageal cancer	49.4 (41.6–57.7)	69.6 (62.7–76.8)	51.9 (48.2–55.5)	34.9 (31.1–38.8)
C.4.4	Gallbladder and biliary tract cancer	11.5 (7.8–15.4)	12.4 (9.6–15.4)	7.1 (5.6–8.6)	4.1 (3–5.3)
C.4.5	Liver cancer	0.7 (0.5–0.9)	4.4 (3.6–5.3)	14.2 (12.1–16.4)	23 (18.5–27.6)
C.4.6	Other and ill‐defined digestive organs	0.5 (0.2–0.8)	1.1 (0.7–1.6)	1.4 (0.9–2)	1.5 (0.7–2.3)
C.4.7	Other and unspecified parts of biliary tract	0.7 (0.4–1)	2.1 (1.4–2.8)	3.6 (2.6–4.6)	4 (2.5–5.6)
C.4.8	Pancreatic cancer	0.5 (0.3–0.6)	3.2 (2.4–4)	11.8 (9.7–14)	20.5 (15.8–25.4)
C.4.9	Rectosigmoid junction	0.8 (0.5–1.1)	3.1 (2.3–3.8)	6.8 (5.5–8.2)	8.9 (6.6–11.3)
C.4.10	Rectum	6.7 (5.2–8.4)	15.5 (13.2–17.8)	19.3 (17.1–21.5)	17.9 (15.1–20.7)
C.4.11	Small Intestine	1.1 (0.7–1.6)	3.5 (2.6–4.4)	5.8 (4.5–7)	6.3 (4.6–8.1)
C.4.12	Stomach cancer	73.3 (62.8–84)	124.7 (114.5–135.1)	114.6 (109.5–119.7)	86.6 (79.4–94)
C.5	**Eye, brain, and other parts of central nervous system**	**1.4 (0.6–2.2)**	**5.7 (4.2–7.3)**	**14.5 (13.6–15.5)**	**22.3 (17.7–26.9)**
C.5.1	Brain and nervous system cancers	0.7 (0.3–1.1)	3.3 (2.4–4.3)	9.4 (8.5–10.5)	14.2 (11–17.6)
C.5.2	Eye and adnexa	0.7 (0.3–1.1)	2 (1.4–2.8)	3 (2.3–3.7)	2.8 (1.8–4)
C.5.3	Meninges	0 (0–0.1)	0.2 (0.1–0.3)	0.7 (0.3–1.1)	1.4 (0.4–2.4)
C.5.4	Spinal cord, cranial nerves, and other parts of central nervous system	0 (0–0)	0.2 (0.1–0.3)	1.4 (1–1.9)	3.9 (2.4–5.5)
C.6	**Female genital organs**	**21.6 (10.9–32.5)**	**47.3 (36–58.7)**	**63.9 (60.4–67.3)**	**67.7 (55–80.4)**
C.6.1	Cervical cancer	9.9 (4.9–15.3)	16.3 (11.9–21)	14.1 (11.9–16.4)	10.8 (8–14)
C.6.2	Corpus uteri	4 (1.9–6.3)	11.9 (8.6–15.5)	20.3 (17.5–23.3)	23.9 (18.2–30.1)
C.6.3	Other and unspecified female genital organs	0.2 (0–0.7)	0.5 (0.1–1)	0.7 (0.2–1.2)	0.7 (0.1–1.5)
C.6.4	Ovarian cancer	4.7 (2.3–7.4)	12.4 (9.1–16.1)	19 (16.5–21.6)	20.6 (15.8–25.8)
C.6.5	Placenta	0.1 (0–0.4)	0.1 (0–0.2)	0.1 (0–0.1)	0 (0–0.1)
C.6.6	Uterine cancer	0.7 (0.2–1.3)	2.5 (1.4–3.7)	5.4 (3.7–7.1)	7.4 (4.4–10.7)
C.6.7	Vagina	0.4 (0.1–0.9)	1.1 (0.5–1.8)	1.8 (1–2.7)	2 (0.8–3.4)
C.6.8	Vulva	1.6 (0.3–3.3)	2.5 (1–4.3)	2.5 (1.2–3.8)	2.1 (0.8–3.7)
C.7	**Leukemia**	**7 (3.9–10)**	**19.1 (15–23.1)**	**31 (29.5–32.5)**	**37 (31–43.1)**
C.8	**Lip, oral cavity, and pharynx**	**18.4 (6.6–30.2)**	**21.7 (14.9–28.7)**	**16.6 (15.4–17.9)**	**12.8 (9.5–16.1)**
C.8.1	Base of Tongue	1.2 (0.1–3.1)	0.8 (0.3–1.6)	0.4 (0.2–0.7)	0.2 (0.1–0.5)
C.8.2	Malignant neoplasm of other and ill‐defined sites in the lip, oral cavity, and pharynx	0.3 (0–0.8)	0.5 (0.2–1)	0.5 (0.2–0.8)	0.4 (0.1–0.7)
C.8.3	Floor of mouth	0.3 (0–0.8)	0.4 (0.1–0.8)	0.3 (0.1–0.6)	0.2 (0–0.5)
C.8.4	Gum	0.4 (0–1.1)	0.7 (0.2–1.2)	0.7 (0.3–1.1)	0.6 (0.2–1.1)
C.8.5	Hypopharynx	0.7 (0.1–1.4)	1 (0.5–1.7)	0.9 (0.5–1.3)	0.6 (0.3–1.1)
C.8.6	Lip	7.2 (2.4–12.6)	4.9 (2.9–7.1)	1.3 (0.9–1.8)	0.4 (0.2–0.7)
C.8.7	Nasopharynx cancer	1.4 (0.4–2.5)	2.2 (1.3–3.2)	1.8 (1.3–2.3)	1.3 (0.8–1.9)
C.8.8	Oropharynx	0.1 (0–0.3)	0.1 (0–0.3)	0.2 (0–0.3)	0.1 (0–0.3)
C.8.9	Other and unspecified parts of mouth	2.8 (0.8–5.3)	3.3 (1.9–5)	2.2 (1.5–2.8)	1.4 (0.8–2)
C.8.10	Other and unspecified parts of tongue	1.6 (0.5–3.1)	3.7 (2.3–5.3)	4.3 (3.4–5.2)	3.8 (2.6–5.2)
C.8.11	Other and unspecified major salivary glands	0.4 (0.1–0.9)	0.7 (0.3–1.2)	0.8 (0.4–1.1)	0.7 (0.3–1.2)
C.8.12	Palate	0.3 (0–0.8)	0.5 (0.2–0.9)	0.5 (0.3–0.8)	0.4 (0.2–0.8)
C.8.13	Parotid gland	0.6 (0.2–1.2)	1.4 (0.8–2.2)	1.7 (1.2–2.2)	1.5 (0.9–2.2)
C.8.14	Pyriform sinus	0.4 (0–1.1)	0.5 (0.1–1)	0.4 (0.1–0.6)	0.2 (0–0.4)
C.8.15	Tonsil	0.6 (0.1–1.3)	0.8 (0.3–1.4)	0.8 (0.5–1.2)	0.7 (0.3–1.2)
C.9	**Lymphoma**	**6.5 (2.6–10.5)**	**9.3 (6.6–12)**	**8 (7.4–8.6)**	**6.4 (4.9–8)**
C.10	**Male genital organs**	**17.9 (10.7–25.3)**	**60.8 (49.3–72.3)**	**125.8 (121.1–130.5)**	**171 (148.7–193.6)**
C.10.1	Other and unspecified male genital organs	0.2 (0–0.4)	0.3 (0.1–0.6)	0.4 (0.1–0.7)	0.3 (0–0.7)
C.10.2	Penis	0.3 (0–0.9)	0.4 (0–0.9)	0.3 (0.1–0.6)	0.2 (0–0.5)
C.10.3	Prostate cancer	16.4 (9.7–23.2)	58.4 (47.3–69.6)	123.6 (118.9–128.4)	169.2 (147.1–191.4)
C.10.4	Testicular cancer	1 (0.4–1.7)	1.6 (0.9–2.4)	1.5 (0.9–2)	1.2 (0.6–1.8)
C.11	**Other and ill‐defined Sites**	**7.7 (0–16)**	**6.9 (3–10.9)**	**3.9 (3.3–4.4)**	**2.4 (1.3–3.5)**
C.12	**Peripheral nerves and autonomic nervous system**	**0 (0–0)**	**0 (0–0)**	**0.1 (0–0.2)**	**1.7 (0–4.8)**
C.13	**Respiratory system and intrathoracic organs**	**18.8 (11.7–26.1)**	**43.4 (35.4–51.4)**	**63.1 (60.5–65.7)**	**69.9 (60.1–79.8)**
C.13.1	Accessory sinuses	0.5 (0.1–0.9)	0.9 (0.5–1.3)	0.9 (0.6–1.3)	0.9 (0.4–1.3)
C.13.2	Heart, mediastinum, and pleura	0.4 (0.2–0.7)	1.2 (0.8–1.7)	2.1 (1.5–2.7)	2.7 (1.6–3.7)
C.13.3	Larynx cancer	7.7 (4.6–11)	13.5 (10.6–16.6)	13.9 (12.1–15.6)	12.2 (9.7–14.8)
C.13.4	Nasal cavity and middle ear	1.6 (0.5–2.9)	1.5 (0.8–2.3)	0.8 (0.5–1.2)	0.5 (0.2–0.8)
C.13.5	Other and ill‐defined sights within respiratory system and intrathoracic organs	0 (0–0)	0 (0–0.1)	0 (0–0.1)	0.1 (0–0.2)
C.13.6	Thymus	0 (0–0.1)	0.1 (0–0.2)	0.2 (0–0.3)	0.2 (0–0.4)
C.13.7	Trachea	0.1 (0–0.4)	0.3 (0–0.6)	0.3 (0.1–0.6)	0.3 (0–0.7)
C.13.8	Bronchus, and lung cancer	8.5 (5.1–12.1)	25.9 (20.8–31)	44.8 (42.1–47.5)	53.1 (45.4–61.1)
C.14	**Retroperitoneum and peritoneum**	**250.2 (97.1–403.8)**	**29.4 (0–60)**	**0.6 (0.4–0.7)**	**0 (0–0.1)**
C.15	**Skin cancer**	**124.8 (100.4–149.3)**	**169.3 (150.5–188.2)**	**136.1 (132.1–140.2)**	**104.4 (93.7–115)**
C.16	**Thyroid and other endocrine glands**	**1.7 (0.6–2.7)**	**5.1 (3.5–6.6)**	**9.9 (9.1–10.6)**	**13.2 (10.1–16.3)**
C.16.1	Adrenal gland	0 (0–0)	0 (0–0.1)	0.5 (0.2–0.7)	1.5 (0.5–2.6)
C.16.2	Other endocrine glands and related structures	0 (0–0)	0.1 (0–0.1)	0.2 (0–0.3)	0.3 (0–0.7)
C.16.3	Thyroid cancer	1.6 (0.6–2.7)	5 (3.5–6.5)	9.2 (8.4–10)	11.4 (8.5–14.3)
C.17	**Urinary tract**	**51.1 (35.2–67.2)**	**78 (65.7–90.4)**	**74.8 (72–77.7)**	**64.3 (56–72.7)**
C.17.1	Bladder cancer	47.8 (32.9–62.7)	69.6 (58.5–80.6)	60.7 (57.9–63.5)	47.3 (40.9–54)
C.17.2	Kidney and other urinary organ cancers	2.1 (1.3–3.1)	6.5 (5.1–8)	11.8 (10.3–13.3)	14.6 (11.9–17.6)
C.17.3	Other and unspecified urinary organs	0.6 (0.1–1.3)	0.8 (0.3–1.4)	0.7 (0.3–1.1)	0.6 (0.2–1)
C.17.4	Renal pelvis	0.3 (0.1–0.5)	0.7 (0.3–1)	1 (0.5–1.5)	1.1 (0.4–1.8)
C.17.5	Ureter	0.2 (0–0.5)	0.4 (0.1–0.8)	0.6 (0.2–1)	0.7 (0.2–1.2)
C.18	**Unknown primary sites**	**1.7 (1–2.5)**	**12.5 (9.8–15.1)**	**56.6 (54–59.2)**	**120.5 (103.2–137.8)**

*Note*: Bold indicates main group of cause.

### 3.2 Both sexes

**FIGURE 3:**
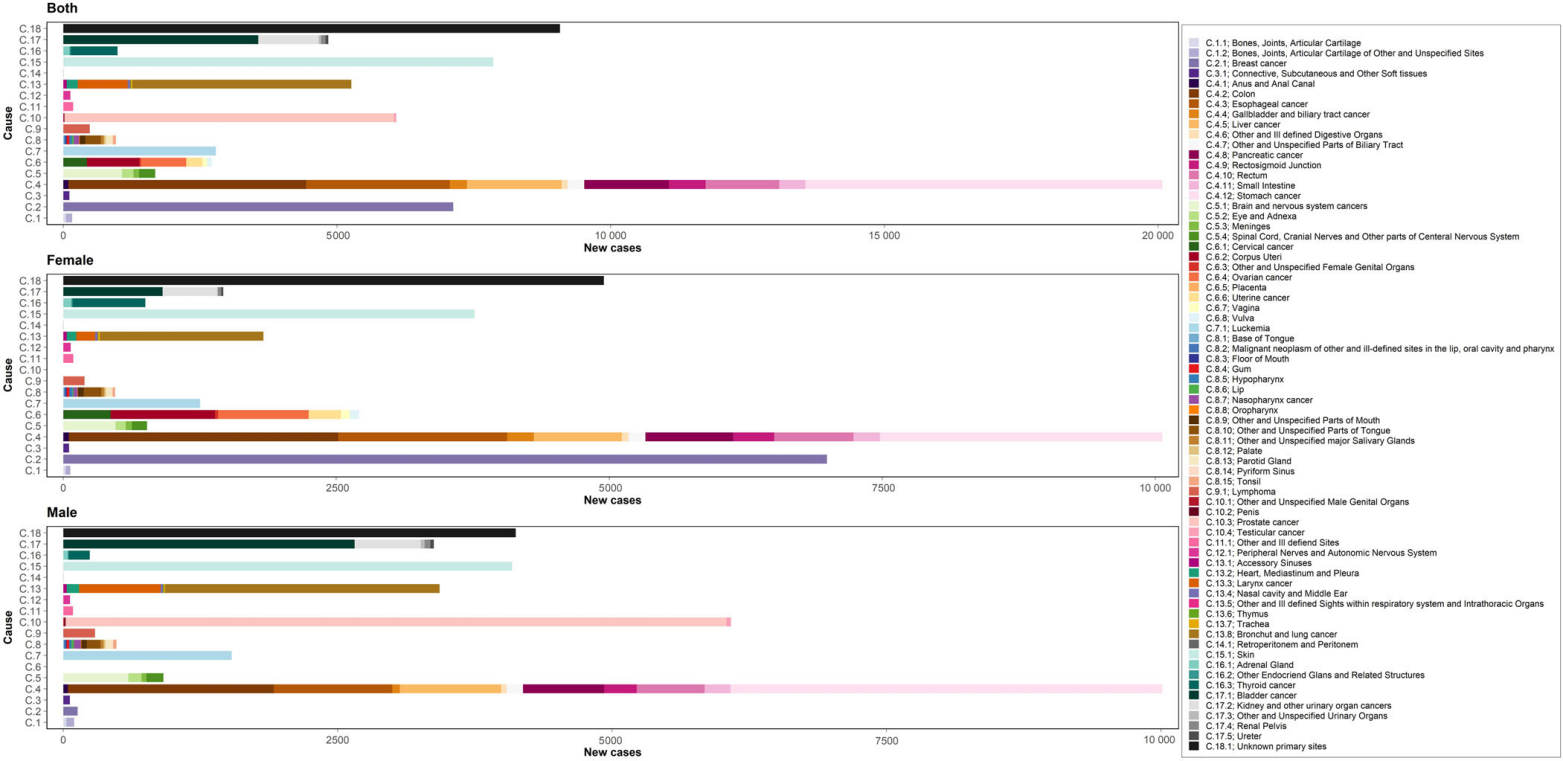
The incidence number of 18 main groups and 70 subgroups of cancers by sex in 2016 in Iran in the elderly population.

Seven incident sites of cancer, according to incidence number, accounted for 50% of all cancers at the national level in both sexes in 2016 and were included in the analysis: skin, breast, stomach, prostate, colon, lung, and bladder cancer. (Figure 3) All the aforementioned subtypes of cancer showed an increasing trend over the study period for both sexes combined, except for the bladder, esophageal, and skin cancers were almost equal in all 27 years.

### 3.3 Cancers in the elderly men

Prostate, skin, stomach, bladder, lung, colon, leukemia, and esophageal cancers were the eight most common cancers (about 67% of all cancers) in elderly men in 2016, according to incidence numbers (Figure 3) and, prostatic cancer incidence number accounted for more than 17% of all cancers among elderly men. All most incident cancers in men had an increasing trend except for esophageal cancer which had a decreasing trend in ASIR (49.4 [41.6–57.7] in 1990 vs. 34.9 [31.1–38.8] per 100 000 in 2016), and skin cancer which remained remarkably constant over time (Table S2).

### 3.4 Cancers in the elderly women

Breast, skin, stomach, colon, esophageal, lung, leukemia, corpus uteri, and bladder cancers were the eight most common cancers among elderly women in 2016, accounting for more than 60% of all cancers, based on incidence number (Figure 3). Breast cancer accounted for more than 19% of all cancers among elderly women as the first and most incident cancer among elderly women (Figure 2). Almost the aforementioned cancers had an increasing trend except for bladder, skin, and esophageal cancers, which were stable in 27 years (Table S3).

### 3.5 Comparison between men and women

All most‐incident cancers were higher among men than in women except for colon cancer which was slightly higher among women than men, with 61.9 (55.6–68.5) and 53.1 (47.6–58.8) per 100 000 in 2016, respectively. Stomach cancer had the third highest ASIR in both men and women, with 110.2 (101.5–119.1) and 65.4 (59.6–71.4) per 100 000 in 2016, respectively. (Tables S2 and S3) Most incident cancers among the oldest old (aged 80+) are the same as in elderly men and women.

### 3.6 Provinces

**FIGURE 4:**
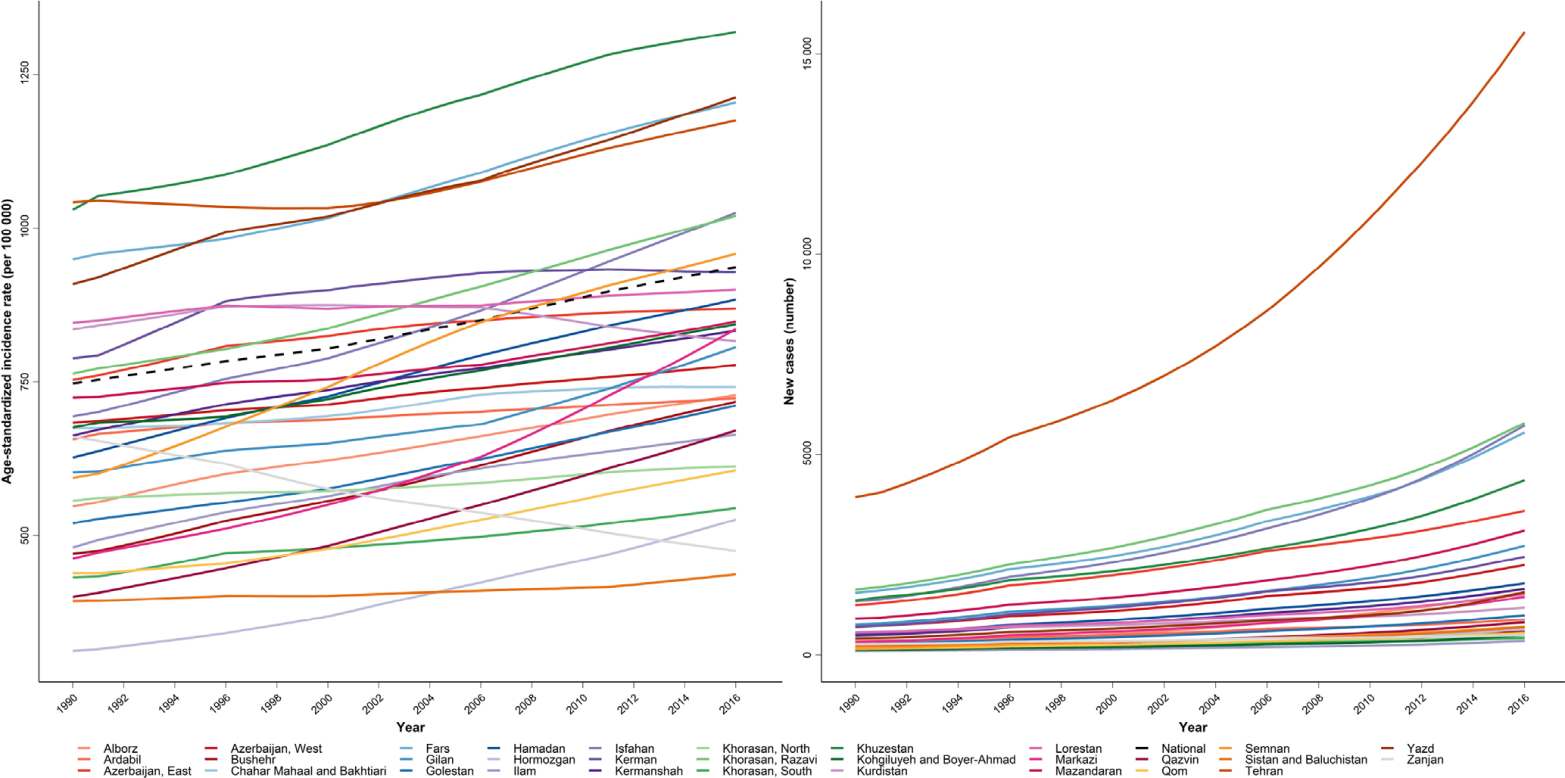
The age‐standardized incidence rate of all cancer combined in the elderly population in all 31 provinces of Iran by year from 1990 to 2016.

At the sub‐national level, there were no significant differences in the Max/Min ratio between provinces in 1990 and 2019. Most provinces had an increasing trend in ASIR from 1990 to 2016 except Zanjan which had a decreasing trend from 660.7 (178.5–2446.0) in 1990 to 475.3 (303.0–745.6) per 100 000 in 2016 in most provinces. However, ASIR was highest in Khuzestan with 1319.2 (1098.3–1584.7) per 100 000 and lowest in Sistan and Baluchistan with 437.3 (365.3–523.6) per 100 000 in 2016 (Figure 4).

The current study showed a significant disparity among provinces of Iran in ASIR of skin cancer ranging from 149.3 (134.4–164.1) in Khuzestan and 34.6 (28.5–40.6) in Zanjan per 100 000. The ASIR of skin cancer was highest in the center located provinces of Iran. The same pattern was seen among men and women (Figure 1).

There was a significant disparity in breast cancer's ASIR with the Max/Min ratio of 5.5, among provinces for all cancer, Tehran with 281.0 (254.3–307.9) per 100 000 had the highest rate, and Zanjan with 51.2 (40.6–61.8) per 100 000 had the lowest rate in 2016.

## 4 DISCUSSION

Herein, we reported the national and sub‐national trends of elderly cancer incidence from 1990 to 2016. We also presented the eight most frequent cancers by sex at national and sub‐national levels. To the best of our knowledge, this is the first study that has examined cancer epidemiology in Iran at the national and sub‐national levels among adults 60 years and older over a period of 27 years from 1990 to 2016. Overall, all cancer combined had an increasing trend in number and rate at the national level, in men, women, and both sexes, and in most provinces. Breast, lung, colon, and stomach cancers were the most incident types among females, and prostate, lung, and colon cancer were the leading types among males, according to incidence number. The male predominance was observed in all leading causes of cancer. Almost all CIR was increasing except bladder, skin, and esophageal cancers from 1990 to 2016. With increasing age, the CIR had an increasing trend in all cancer combined.

As the main result of this study, cancer incidence had an increasing trend over 27 years from 1990 to 2016, an important part of this increase could be a consequence of the growing, and aging population.^19^ Our results demonstrated the proportion of all cancers among people of all ages that occurred in the elderly was 55.5%. Older individuals are more likely to develop cancer as a result of prolonged exposure to carcinogenic agents, accumulation of DNA damage, mutations of tumor suppressor genes, oncogenic activation, and diminished immune response.^20^ On the other hand, we expect that with the quantitative and qualitative progress of preventive and control cares and interventions, the onset of cancer in people will be delayed, and as a result, instead of observing cancer in young and middle‐aged people, the percentage of cancer incidence in the elderly will constantly increase compared to other age groups.^21^

We showed prostate, skin, stomach, bladder, lung, colon, leukemia, and esophageal cancers in elderly men, and breast, skin, stomach, colon, esophageal, lung, leukemia, corpus uteri, and bladder cancers among elderly women were the most common cancers, accounting for more than half of all cancers. These findings were compatible with the results of a study in 2008 in Tehran.^9^ However, in a study published in European countries, prostate cancer, lung cancer, and colon cancer in men, and breast cancer, colon cancer, and lung cancer in women accounted for approximately half of all cancer cases diagnosed over 65 years of age.^22^ A study in 2012 showed five leading cancer types among males aged 65 years and older in Middle East and Northern Africa unlike our study liver was one of the leading cancers.^1^

There was almost always a greater ASIR of skin cancer among males compared to females, it may be attributable to their prolonged exposure to solar radiation, smoking, and outdoor work due to their longer exposure to ultraviolet rays.^23^, ^24^ Current study showed a significant disparity among provinces of Iran. The factors contributing to differences in skin cancer among provinces include sunlight exposure, infectious skin conditions, poor hygiene, and an insufficient water supply, in addition to sociodemographic predictors such as public health insurance access and socioeconomic status.^25^ Aging is undeniably a major risk factor associated with skin cancer,^26^ as the result showed us CIR of skin cancer increased with aging.

In the current study, the incidence rate of prostate cancer increased from 1990 to 2016. This increasing trend of prostate cancer incident could be the result of increasing smoking behaviors,^27^ elderly population growing,^28^ and high trans‐fatty acids intake^29^ in the Iran population. Generally, with the advent of prostate‐specific antigen screening and an increased life expectancy, a growing number of elderly men are being diagnosed with prostate cancer.^30^

This study showed breast cancer is the first and most incident cancer among women and demonstrated that had a significant increasing trend in 27 years. According to a case–control study conducted in Iran, obese women were three times more likely to develop breast cancer than those without obesity regardless of age, age at menopause, family history, and parity.^31^ It has been shown over the past three decades, prevalence of overweight and obesity have both increased considerably in Iran.^32^ Albeit, increasing in smoking behavior could have a role in this increasing trend of CIR of breast cancer.^27^ Several of the factors that might be contributed to the increase in breast cancer incidence: improvements in the INCR system and an increase in the number of women registered in this system, enhancements in diagnostic tools, greater healthcare coverage, as well as increased awareness of breast cancer symptoms by the general population and a more positive attitude toward mammograms despite cultural barriers.^16^ Our findings demonstrated a considerable provincial disparity in breast cancer incidence, with Tehran having the highest rate. The results may reflect a variation in awareness and acceptance of screening in small provinces compared to larger cities, like Tehran, which has more advanced diagnostic facilities, and oncologists, in addition to a higher level of education among patients.^16^

There was a higher CIR in men than in women with bladder cancer. A number of environmental factors may contribute to bladder cancer, including exposure to chemical components in industrial processes and natural arsenic contamination.^33^, ^34^ According to a study conducted in Iran, about 90% of employed women are employed in service sectors, compared to about 21% of men. Most men work in agriculture and the manufacturing industry, which exposes them to more occupational carcinogens.^35^ This could put men more exposure to chemical components of industrial processes and natural arsenic contamination and following more risk of bladder cancer. On the other hand, smoking is known to be a risk factor for bladder cancer^36^ and it is increasing behavior among Iranian.^27^

Lung cancer had an increasing trend in men and women, and incidence rates of lung cancer were twice as high in men as in women. Overall, these findings are in accordance with findings reported in a study about lung cancer at the national and sub‐national levels.^15^ This difference between men and women could explain by the prevalence of smoking is higher in men than in women.^37^ As smoking is more prevalent among men than women, women are at a high risk of secondhand smoking.^38^ Meanwhile, air pollution is increasing in developing countries such as Iran due to the activities of industrial production and rising emissions from vehicles, and outdoor air pollution is believed to be a contributing factor to the risk of lung cancer.^39^

In general, the increased number of elderly patients with cancer will pose a great challenge to the entire healthcare system. Consequently, it is imperative to implement comprehensive cancer control strategies for this age group. Additionally, clinical research is needed to identify and implement evidence‐based, best practices to improve the quality of life of older cancer patients in addition to eliminating their suffering and extending their meaningful lives. Identifying specific risk factors that may contribute to the increase in cancer incidence among the elderly can be the next step. As an example, research could explore the effects of tobacco use, dietary habits, sedentary lifestyles, and exposure to carcinogens specifically in the elderly.^40^ Meanwhile, healthcare providers and the elderly must receive appropriate education about screening and prevention and offer optimal care to older cancer patients. Health policymakers may develop structured training programs and incentives to encourage healthcare workers to pursue academic careers in geriatric medicine and integrate geriatric concepts into training programs in oncology. A policymaker must be aware of this problem and allocate funds and programs to support the care of older cancer patients.

## 5 CONCLUSION

We have reported an increasing pattern of incidence number, rate, and ASIR in all cancers combined and all most incident cancer subtypes in both sexes at the national and almost all provinces from 1990 to 2016. Regarding the persistent increasing trend of most elderly cancer incidence, this is crucial for policymakers to establish preventive plans, determine proper resource allocation, and develop specific strategies for the treatment of elderly cancer patients for elderly cancers.

### 5.1 Limitations of the study

There was a limitation of this study in that there was no information on the staging of all cancers, data on risk‐attributed incidence, cancer prognosis, and surveillance in Iran. We also encountered a limitation in this study regarding the method of addressing the incompleteness via the SSO cancer registry as data on a small percentage of patients not supported by SSO are still missing.

## AUTHOR CONTRIBUTIONS

**Zahra Shokri Varniab:** Data curation (equal); investigation (equal); project administration (equal); validation (equal); writing – original draft (equal); writing – review and editing (equal). **Sahar Saeedi Moghaddam:** Investigation (equal); software (equal); validation (equal); visualization (equal); writing – review and editing (equal). **Ashkan Pourabhari Langroudi:** Data curation (equal); validation (equal); writing – original draft (equal); writing – review and editing (equal). **Sina Azadnajafabad:** Data curation (equal); writing – review and editing (equal). **Seyede Salehe Mortazavi:** Conceptualization (equal); writing – review and editing (equal). **Ali Sheidaei:** Data curation (equal); formal analysis (equal); software (equal). **Kimiya Gohari:** Investigation (equal); methodology (equal). **Yosef Farzi:** Methodology (equal). **Zeinab Shirzad Moghaddam:** Methodology (equal). **Hanye Sohrabi:** Methodology (equal). **Mohsen Shati:** Conceptualization (equal); project administration (equal); visualization (equal).

## FUNDING INFORMATION

This study was funded by National Institute for Medical Research Development (NIMAD) (grant number of 963 348). We declare that the funding bodies had no role in the design of the study and collection, analysis, and interpretation of data and in writing the manuscript.

## CONFLICT OF INTEREST STATEMENT

The authors have stated explicitly that there are no conflicts of interest in connection with this article.

## ETHICS STATEMENT

The Ethics Committee of the National Institute for Medical Research Development (NIMAD) approved the study protocol (IR.NIMAD.REC.1396.192).

## Supporting information


**Table S1.** Cancer codes.

**Table S2.** The age‐standardized incidence rate of 18 main groups and 70 subgroups of cancers in elderly men between 1990 and 2016 in Iran.

**Table S3.** The age‐standardized incidence rate of 18 main groups and 70 subgroups of cancers in elderly women between 1990 and 2016 in Iran.

## Data Availability

Data will be shared, upon the request.
