# Supplementary material for: The levels and trends of cancer incidence in the elderly population at national and sub‐national scales in Iran from 1990 to 2016
Source: Cancer Rep (Hoboken). 2023 Dec 4;7(1):e1937. doi: 10.1002/cnr2.1937 (PMC10809202; doi:10.1002/cnr2.1937)
Supplement: Supplementary file 1 — Table S1. Cancer codes. [file CNR2-7-e1937-s001.docx]

| ***Cause code*** | ***Cause label*** |
| --- | --- |
| C.1 | Bones, joints, and articular cartilage |
| C.1.1 | Bones, joints, articular cartilage of limbs |
| C.1.2 | Bones, joints, articular cartilage of other and unspecified sites |
| C.2 | Breast |
| C.3 | Connective, subcutaneous, and other soft tissues |
| C.4 | Digestive organs |
| C.4.1 | Anus and anal canal |
| C.4.2 | Colon |
| C.4.3 | Esophageal |
| C.4.4 | Gallbladder and biliary tract |
| C.4.5 | Liver |
| C.4.6 | Other and ill-defined digestive organs |
| C.4.7 | Other and unspecified parts of biliary tract |
| C.4.8 | Pancreatic |
| C.4.9 | Rectosigmoid junction |
| C.4.10 | Rectum |
| C.4.11 | Small intestine |
| C.4.12 | Stomach |
| C.5 | Eye, brain, and other parts of central nervous system |
| C.5.1 | Brain and nervous system |
| C.5.2 | Eye and adnexa |
| C.5.3 | Meninges |
| C.5.4 | Spinal cord, cranial nerves, and other parts of central nervous system |
| C.6 | Female genital organs |
| C.6.1 | Cervical |
| C.6.2 | Corpus uteri |
| C.6.3 | Other and unspecified female genital organs |
| C.6.4 | Ovarian |
| C.6.5 | Placenta |
| C.6.6 | Uterine |
| C.6.7 | Vagina |
| C.6.8 | Vulva |
| C.7 | Leukemia |
| C.8 | Lip, oral cavity, and pharynx |
| C.8.1 | Base of tongue |
| C.8.2 | Malignant neoplasm of other and ill-defined sites in the lip, oral cavity, and pharynx |
| C.8.3 | Floor of mouth |
| C.8.4 | Gum |
| C.8.5 | Hypopharynx |
| C.8.6 | Lip |
| C.8.7 | Nasopharynx cancer |
| C.8.8 | Oropharynx |
| C.8.9 | Other and unspecified parts of mouth |
| C.8.10 | Other and unspecified parts of tongue |
| C.8.11 | Other and unspecified major salivary glands |
| C.8.12 | Palate |
| C.8.13 | Parotid gland |
| C.8.14 | Pyriform sinus |
| C.8.15 | Tonsil |
| C.9 | Lymph nodes |
| C.10 | Male genital organs |
| C.10.1 | Other and unspecified male genital organs |
| C.10.2 | Penis |
| C.10.3 | Prostate |
| C.10.4 | Testicular |
| C.11 | Malignant neoplasms of ill-defined, secondary, and unspecified sites |
| C.12 | Peripheral nerves and autonomic nervous system |
| C.13 | Respiratory system and intrathoracic organs |
| C.13.1 | Accessory sinuses |
| C.13.2 | Heart, mediastinum, and pleura |
| C.13.3 | Larynx |
| C.13.4 | Nasal cavity and middle ear |
| C.13.5 | Other and ill-defined sights within respiratory system and intrathoracic organs |
| C.13.6 | Thymus |
| C.13.7 | Trachea |
| C.13.8 | Bronchus, and lung |
| C.14 | Retroperitoneum and peritoneum |
| C.15 | Skin |
| C.16 | Thyroid and other endocrine glands |
| C.16.1 | Adrenal gland |
| C.16.2 | Other endocrine glands and related structures |
| C.16.3 | Thyroid |
| C.17 | Urinary tract |
| C.17.1 | Bladder |
| C.17.2 | Kidney and other urinary organs |
| C.17.3 | Other and unspecified urinary organs |
| C.17.4 | Renal pelvis |
| C.17.5 | Ureter |
| C.18 | Unknown primary site |
| **Total All Cancers** | **All cancers** |
