# Supplementary material for: The levels and trends of cancer incidence in the elderly population at national and sub‐national scales in Iran from 1990 to 2016
Source: Cancer Rep (Hoboken). 2023 Dec 4;7(1):e1937. doi: 10.1002/cnr2.1937 (PMC10809202; doi:10.1002/cnr2.1937)
Supplement: Supplementary file 2 — Table S2. The age‐standardized incidence rate of 18 main groups and 70 subgroups of cancers in elderly men between 1990 and 2016 in Iran. [file CNR2-7-e1937-s002.docx]

| **Cause** **code** **Cause** **name** | | Year | | | |
| --- | --- | --- | --- | --- | --- |
|  |  | 1990 | 2000 | 2010 | 2016 |
| Total All Cancers | **All** **Cancers** | **825.1** **(517.6** **to** **1337.1)** | **867.6** **(682.4** **to** **1105.8)** | **938.3** **(852** **to** **1033.3)** | **988.1** **(811.1** **to** **1205)** |
| C.1 | **Bones,** **Joints,** **and** **Articular** **cartilage** | **1** **(0** **to** **2.1)** | **2.1** **(1.1** **to** **3.2)** | **2.7** **(2.4** **to** **3.1)** | **2.8** **(1.7** **to** **4)** |
| C.1.1 | Bones, Joints, and Articular Cartilage | 0.3 (0 to 0.6) | 0.6 (0.3 to 1) | 0.8 (0.6 to 1) | 0.8 (0.4 to 1.3) |
| C.1.2 | Bones, Joints, and Articular Cartilage of Other and Unspecified Sites | 0.7 (0 to 1.5) | 1.5 (0.8 to 2.3) | 2 (1.6 to 2.3) | 2 (1.2 to 2.9) |
| C.2 | **Breast** **cancer** | **1** **(0.7** **to** **1.4)** | **2.3** **(1.8** **to** **2.7)** | **3.3** **(3** **to** **3.6)** | **3.7** **(3.2** **to** **4.3)** |
| C.3 | **Connective** **,** **Subcutaneous** **and** **Other** **Soft** **tissues** | **68.6** **(18.4** **to** **119.1)** | **25.3** **(14.6** **to** **36)** | **5.1** **(4.6** **to** **5.6)** | **1.7** **(1.1** **to** **2.3)** |
| C.4 | **Digestive** **organs** | **173.9** **(151.5** **to** **196.9)** | **297.1** **(276.5** **to** **318.1)** | **309.6** **(303.8** **to** **315.4)** | **281.7** **(264.1** **to** **299.4)** |
| C.4.1 | Anus and Anal Canal | 1.8 (0.8 to 2.8) | 2.4 (1.5 to 3.4) | 1.8 (1.2 to 2.4) | 1.2 (0.7 to 1.8) |
| C.4.2 | Colon cancer | 9.2 (7.5 to 11) | 28.9 (25.8 to 32.2) | 48.5 (45.2 to 52) | 53.1 (47.6 to 58.8) |
| C.4.3 | Esophageal cancer | 51.2 (43.3 to 59.5) | 67.1 (60.6 to 73.9) | 46.9 (43.5 to 50.3) | 30.2 (26.9 to 33.7) |
| C.4.4 | Gallbladder and biliary tract cancer | 7 (4.6 to 9.5) | 6.7 (5.1 to 8.3) | 3.5 (2.7 to 4.2) | 1.9 (1.3 to 2.4) |
| C.4.5 | Liver cancer | 0.9 (0.6 to 1.2) | 5.3 (4.3 to 6.3) | 16.2 (13.7 to 18.6) | 25.9 (20.9 to 31.1) |
| C.4.6 | Other and Ill‐defined Digestive Organs | 0.5 (0.2 to 0.8) | 1.1 (0.7 to 1.6) | 1.4 (0.9 to 2) | 1.4 (0.7 to 2.2) |
| C.4.7 | Other and Unspecified Parts of Biliary Tract | 0.8 (0.4 to 1.1) | 2.3 (1.5 to 3) | 3.8 (2.7 to 4.8) | 4.2 (2.6 to 5.8) |
| C.4.8 | Pancreatic cancer | 0.5 (0.3 to 0.7) | 3.3 (2.5 to 4.1) | 12.1 (9.9 to 14.3) | 20.9 (16 to 25.8) |
| C.4.9 | Rectosigmoid Junction | 0.8 (0.5 to 1.1) | 3 (2.2 to 3.7) | 6.5 (5.2 to 7.7) | 8.4 (6.2 to 10.7) |
| C.4.10 | Rectum | 7.1 (5.5 to 8.8) | 15.7 (13.5 to 18.1) | 19.1 (17 to 21.3) | 17.5 (14.9 to 20.3) |
| C.4.11 | Small Intestine | 1.3 (0.8 to 1.8) | 3.9 (2.9 to 4.9) | 6.1 (4.9 to 7.4) | 6.6 (4.8 to 8.5) |
| C.4.12 | Stomach cancer | 93 (80.4 to 105.8) | 157.3 (145.2 to 169.7) | 143.7 (138 to 149.6) | 110.2 (101.5 to 119.1) |
| C.5 | **Eye,** **brain** **and** **other** **parts** **of** **central** **nervous** **system** | **1.7** **(0.7** **to** **2.6)** | **6.9** **(5** **to** **8.7)** | **17.1** **(16.1** **to** **18.2)** | **26.1** **(20.8** **to** **31.5)** |
| C.5.1 | Brain and nervous system cancers | 0.8 (0.3 to 1.3) | 4 (2.9 to 5.2) | 11.3 (10.2 to 12.5) | 17.1 (13.2 to 21.1) |
| C.5.2 | Eye and Adnexa | 0.8 (0.3 to 1.3) | 2.4 (1.7 to 3.3) | 3.5 (2.7 to 4.4) | 3.4 (2.1 to 4.8) |
| C.5.3 | Meninges | 0 (0 to 0.1) | 0.2 (0.1 to 0.3) | 0.7 (0.3 to 1) | 1.3 (0.4 to 2.3) |
| C.5.4 | Spinal Cord, Cranial Nerves, and Other parts of Central Nervous System | 0 (0 to 0) | 0.2 (0.1 to 0.3) | 1.6 (1.1 to 2.1) | 4.4 (2.7 to 6.3) |
| C.7 | **Leukemia** | **8.9** **(5** **to** **12.8)** | **23.7** **(18.7** **to** **28.6)** | **37.1** **(35.3** **to** **38.8)** | **43.6** **(36.5** **to** **50.7)** |
| C.8 | **Lip,** **oral** **cavity** **and** **pharynx** | **20.4** **(7.4** **to** **33.5)** | **23.7** **(16.3** **to** **31.3)** | **18** **(16.7** **to** **19.2)** | **13.8** **(10.3** **to** **17.3)** |
| C.8.1 | Base of Tongue | 1.2 (0.1 to 2.9) | 0.8 (0.3 to 1.5) | 0.4 (0.2 to 0.6) | 0.2 (0.1 to 0.5) |
| C.8.2 | Malignant neoplasm of other and ill‐defined sites in the lip, oral cavity and pharynx | 0.3 (0 to 0.9) | 0.6 (0.2 to 1) | 0.5 (0.2 to 0.8) | 0.4 (0.1 to 0.7) |
| C.8.3 | Floor of Mouth | 0.3 (0 to 0.7) | 0.4 (0.1 to 0.7) | 0.3 (0.1 to 0.5) | 0.2 (0 to 0.5) |
| C.8.4 | Gum | 0.5 (0 to 1.1) | 0.7 (0.2 to 1.2) | 0.7 (0.3 to 1.1) | 0.7 (0.2 to 1.2) |
| C.8.5 | Hypopharynx | 0.6 (0.1 to 1.3) | 0.9 (0.4 to 1.5) | 0.8 (0.4 to 1.1) | 0.6 (0.2 to 1) |
| C.8.6 | Lip | 9 (3.1 to 15.6) | 6.6 (4 to 9.6) | 1.9 (1.3 to 2.5) | 0.7 (0.3 to 1) |
| C.8.7 | Nasopharynx cancer | 1.7 (0.5 to 3.1) | 2.7 (1.6 to 4) | 2.4 (1.8 to 3) | 1.8 (1.1 to 2.5) |
| C.8.8 | Oropharynx | 0.1 (0 to 0.3) | 0.2 (0 to 0.4) | 0.2 (0 to 0.4) | 0.2 (0 to 0.4) |
| C.8.9 | Other and Unspecified Parts of Mouth | 2.8 (0.8 to 5.3) | 3.3 (1.9 to 4.9) | 2.2 (1.5 to 2.8) | 1.4 (0.8 to 2.1) |
| C.8.10 | Other and Unspecified Parts of Tongue | 1.4 (0.4 to 2.6) | 3.1 (1.9 to 4.5) | 3.9 (3 to 4.8) | 3.5 (2.3 to 4.9) |
| C.8.11 | Other and Unspecified major Salivary Glands | 0.4 (0.1 to 1) | 0.7 (0.3 to 1.2) | 0.9 (0.5 to 1.2) | 0.8 (0.4 to 1.3) |
| C.8.12 | Palate | 0.4 (0 to 0.9) | 0.5 (0.2 to 1) | 0.6 (0.3 to 0.8) | 0.5 (0.2 to 0.8) |
| C.8.13 | Parotid Gland | 0.7 (0.2 to 1.4) | 1.6 (0.9 to 2.4) | 1.9 (1.3 to 2.5) | 1.7 (1 to 2.5) |
| C.8.14 | Pyriform Sinus | 0.6 (0 to 1.5) | 0.7 (0.2 to 1.3) | 0.5 (0.2 to 0.8) | 0.3 (0.1 to 0.6) |
| C.8.15 | Tonsil | 0.6 (0.1 to 1.4) | 0.9 (0.4 to 1.5) | 1 (0.6 to 1.4) | 0.9 (0.4 to 1.4) |
| C.9 | **Lymphoma** | **8.4** **(3.3** **to** **13.5)** | **12** **(8.5** **to** **15.4)** | **10.3** **(9.5** **to** **11)** | **8.3** **(6.4** **to** **10.3)** |
| C.10 | **Male** **genital** **organs** | **17.9** **(10.7** **to** **25.3)** | **60.8** **(49.3** **to** **72.3)** | **125.8** **(121.1** **to** **130.5)** | **171** **(148.7** **to** **193.6)** |
| C.10.1 | Other and Unspecified Male Genital Organs | 0.2 (0 to 0.4) | 0.3 (0.1 to 0.6) | 0.4 (0.1 to 0.7) | 0.3 (0 to 0.7) |
| C.10.2 | Penis | 0.3 (0 to 0.9) | 0.4 (0 to 0.9) | 0.3 (0.1 to 0.6) | 0.2 (0 to 0.5) |
| C.10.3 | Prostate cancer | 16.4 (9.7 to 23.2) | 58.4 (47.3 to 69.6) | 123.6 (118.9 to 128.4) | 169.2 (147.1 to 191.4) |
| C.10.4 | Testicular cancer | 1 (0.4 to 1.7) | 1.6 (0.9 to 2.4) | 1.5 (0.9 to 2) | 1.2 (0.6 to 1.8) |
| C.11 | **Other** **and** **Ill‐defined** **Sites** | **8.6** **(0** **to** **17.9)** | **7.6** **(3.3** **to** **11.8)** | **4.1** **(3.6** **to** **4.6)** | **2.5** **(1.4** **to** **3.7)** |
| C.12 | **Peripheral** **Nerves** **and** **Autonomic** **Nervous** **System** | **0** **(0** **to** **0)** | **0** **(0** **to** **0)** | **0.1** **(0** **to** **0.2)** | **1.8** **(0** **to** **5)** |
| C.13 | **Respiratory** **system** **and** **Intrathoracic** **organs** | **26.1** **(16.3** **to** **35.9)** | **59.3** **(48.6** **to** **70.1)** | **86.1** **(82.9** **to** **89.4)** | **96.8** **(83.7** **to** **110.3)** |
| C.13.1 | Accessory Sinuses | 0.5 (0.2 to 1) | 1 (0.5 to 1.4) | 1 (0.6 to 1.4) | 0.9 (0.4 to 1.4) |
| C.13.2 | Heart, Mediastinum, and Pleura | 0.5 (0.2 to 0.9) | 1.5 (1 to 2.1) | 2.6 (1.8 to 3.3) | 3.2 (2 to 4.5) |
| C.13.3 | Larynx cancer | 11.9 (7.2 to 16.8) | 21.2 (16.8 to 25.9) | 22.8 (20.1 to 25.5) | 21 (16.9 to 25.5) |
| C.13.4 | Nasal cavity and Middle Ear | 1.8 (0.6 to 3.4) | 1.7 (0.9 to 2.6) | 0.9 (0.5 to 1.3) | 0.5 (0.3 to 0.8) |
| C.13.5 | Other and Ill defined Sights within respiratory system and Intrathoracic Organs | 0 (0 to 0) | 0 (0 to 0.1) | 0 (0 to 0.1) | 0.1 (0 to 0.3) |
| C.13.6 | Thymus | 0 (0 to 0.1) | 0.1 (0 to 0.3) | 0.2 (0.1 to 0.4) | 0.3 (0 to 0.5) |
| C.13.7 | Trachea | 0.1 (0 to 0.3) | 0.2 (0 to 0.5) | 0.3 (0.1 to 0.5) | 0.3 (0 to 0.6) |
| C.13.8 | Bronchus, and lung cancer | 11.1 (6.8 to 15.7) | 33.5 (27.1 to 40.1) | 58.3 (54.9 to 61.8) | 70.5 (60.4 to 80.9) |
| C.14 | **Retroperitonem** **and** **Peritonem** | **272.3** **(113.4** **to** **431.7)** | **32.5** **(0** **to** **66.2)** | **0.6** **(0.5** **to** **0.8)** | **0.1** **(0** **to** **0.1)** |
| C.15 | **Skin** **cancer** | **140.8** **(114.4** **to** **167.2)** | **187.9** **(167.6** **to** **208.4)** | **149.9** **(145.7** **to** **154)** | **115.2** **(103.7** **to** **126.7)** |
| C.16 | **Thyroid** **and** **other** **endocrine** **glands** | **1** **(0.4** **to** **1.6)** | **2.9** **(2** **to** **3.8)** | **5.4** **(4.9** **to** **5.8)** | **6.9** **(5.3** **to** **8.6)** |
| C.16.1 | Adrenal Gland | 0 (0 to 0) | 0 (0 to 0.1) | 0.4 (0.2 to 0.6) | 1.1 (0.4 to 2) |
| C.16.2 | Other Endocrine Glands and Related Structures | 0 (0 to 0) | 0.1 (0 to 0.2) | 0.2 (0.1 to 0.4) | 0.3 (0 to 0.7) |
| C.16.3 | Thyroid cancer | 1 (0.4 to 1.6) | 2.8 (2 to 3.7) | 4.8 (4.3 to 5.3) | 5.5 (4 to 7.1) |
| C.17 | **Urinary** **tract** | **72.6** **(50.9** **to** **94.7)** | **111** **(94.1** **to** **128)** | **107.8** **(104.1** **to** **111.6)** | **95.3** **(83.2** **to** **107.4)** |
| C.17.1 | Bladder cancer | 68.6 (48 to 89.2) | 101 (85.5 to 116.5) | 91.3 (87.5 to 95) | 74.8 (64.9 to 84.8) |
| C.17.2 | Kidney and other urinary organ cancers | 2.4 (1.5 to 3.4) | 7.2 (5.6 to 8.8) | 13.3 (11.6 to 15.1) | 17.1 (13.8 to 20.7) |
| C.17.3 | Other and Unspecified Urinary Organs | 1 (0.1 to 2.1) | 1.4 (0.5 to 2.3) | 1.2 (0.6 to 1.9) | 1 (0.3 to 1.8) |
| C.17.4 | Renal Pelvis | 0.4 (0.1 to 0.7) | 0.8 (0.4 to 1.3) | 1.3 (0.7 to 1.9) | 1.5 (0.5 to 2.4) |
| C.17.5 | Ureter | 0.3 (0 to 0.6) | 0.5 (0.2 to 1) | 0.8 (0.3 to 1.3) | 0.9 (0.2 to 1.6) |
| C.18 | **Unknown** **Primary** **Sites** | **1.8** **(1** **to** **2.6)** | **12.6** **(9.9** **to** **15.2)** | **55.4** **(53** **to** **57.8)** | **116.6** **(99.8** **to** **133.4)** |
