# Supplementary material for: The levels and trends of cancer incidence in the elderly population at national and sub‐national scales in Iran from 1990 to 2016
Source: Cancer Rep (Hoboken). 2023 Dec 4;7(1):e1937. doi: 10.1002/cnr2.1937 (PMC10809202; doi:10.1002/cnr2.1937)
Supplement: Supplementary file 3 — Table S3. The age‐standardized incidence rate of 18 main groups and 70 subgroups of cancers in elderly women between 1990 and 2016 in Iran. [file CNR2-7-e1937-s003.docx]

| Cause code | Cause name | Year | | | |
| --- | --- | --- | --- | --- | --- |
|  |  | **1990** | **2000** | **2010** | **2016** |
| Total All Cancers | **All** **Cancers** | **655.6** **(411.4** **to** **1060.8)** | **734** **(576.7** **to** **936.4)** | **836.8** **(760** **to** **921.4)** | **889.7** **(731.3** **to** **1083.6)** |
| C.1 | **Bones,** **Joints,** **and** **Articular** **cartilage** | **0.5** **(0** **to** **1.1)** | **1.1** **(0.6** **to** **1.7)** | **1.5** **(1.3** **to** **1.7)** | **1.6** **(1** **to** **2.3)** |
| C.1.1 | Bones, Joints, and Articular Cartilage | 0.1 (0 to 0.3) | 0.3 (0.2 to 0.5) | 0.5 (0.4 to 0.6) | 0.5 (0.3 to 0.8) |
| C.1.2 | Bones, Joints, and Articular Cartilage of Other and Unspecified Sites | 0.4 (0 to 0.8) | 0.8 (0.4 to 1.2) | 1 (0.9 to 1.2) | 1.1 (0.6 to 1.6) |
| C.2 | **Breast** **cancer** | **47** **(32.9** **to** **61.1)** | **108.9** **(93.5** **to** **124.4)** | **157.5** **(152.4** **to** **162.7)** | **174.4** **(155.9** **to** **192.9)** |
| C.3 | **Connective** **,** **Subcutaneous** **and** **Other** **Soft** **tissues** | **48.9** **(11.3** **to** **86.4)** | **18.3** **(10.5** **to** **26.2)** | **3.8** **(3.4** **to** **4.3)** | **1.3** **(0.8** **to** **1.8)** |
| C.4 | **Digestive** **organs** | **135.6** **(116** **to** **156)** | **245.9** **(227.1** **to** **265)** | **271.9** **(266** **to** **277.8)** | **253.5** **(236.9** **to** **270.4)** |
| C.4.1 | Anus and Anal Canal | 1.6 (0.7 to 2.6) | 2.4 (1.4 to 3.3) | 1.9 (1.2 to 2.5) | 1.3 (0.7 to 1.9) |
| C.4.2 | Colon cancer | 9.8 (7.9 to 11.8) | 32.6 (29 to 36.4) | 56.5 (52.6 to 60.4) | 61.9 (55.6 to 68.5) |
| C.4.3 | Esophageal cancer | 47.2 (39.4 to 55.4) | 72.3 (65.1 to 79.9) | 57 (53.1 to 60.9) | 39.1 (34.9 to 43.4) |
| C.4.4 | Gallbladder and biliary tract cancer | 16.5 (11.4 to 22.1) | 18.7 (14.5 to 23) | 10.8 (8.5 to 13.1) | 6.2 (4.5 to 7.9) |
| C.4.5 | Liver cancer | 0.5 (0.3 to 0.7) | 3.5 (2.8 to 4.2) | 12.1 (10.3 to 14) | 20.2 (16.2 to 24.3) |
| C.4.6 | Other and Ill‐defined Digestive Organs | 0.4 (0.2 to 0.7) | 1.1 (0.6 to 1.6) | 1.5 (0.9 to 2) | 1.5 (0.7 to 2.4) |
| C.4.7 | Other and Unspecified Parts of Biliary Tract | 0.6 (0.3 to 0.9) | 2 (1.3 to 2.6) | 3.4 (2.4 to 4.4) | 3.9 (2.4 to 5.4) |
| C.4.8 | Pancreatic cancer | 0.4 (0.3 to 0.6) | 3 (2.3 to 3.8) | 11.6 (9.5 to 13.7) | 20.2 (15.6 to 25) |
| C.4.9 | Rectosigmoid Junction | 0.8 (0.5 to 1.1) | 3.2 (2.4 to 4) | 7.2 (5.8 to 8.7) | 9.4 (6.9 to 12) |
| C.4.10 | Rectum | 6.3 (4.8 to 8) | 15.2 (12.9 to 17.5) | 19.5 (17.3 to 21.7) | 18.2 (15.4 to 21.1) |
| C.4.11 | Small Intestine | 1 (0.6 to 1.4) | 3.1 (2.3 to 3.9) | 5.4 (4.2 to 6.6) | 6.1 (4.4 to 7.8) |
| C.4.12 | Stomach cancer | 50.4 (42.4 to 58.6) | 88.9 (80.8 to 97.1) | 85 (80.6 to 89.4) | 65.4 (59.6 to 71.4) |
| C.5 | **Eye,** **brain** **and** **other** **parts** **of** **central** **nervous** **system** | **1** **(0.5** **to** **1.6)** | **4.5** **(3.3** **to** **5.7)** | **12** **(11.2** **to** **12.8)** | **19** **(15.1** **to** **23)** |
| C.5.1 | Brain and nervous system cancers | 0.5 (0.2 to 0.8) | 2.6 (1.9 to 3.4) | 7.6 (6.8 to 8.5) | 11.8 (9.1 to 14.7) |
| C.5.2 | Eye and Adnexa | 0.5 (0.2 to 0.8) | 1.6 (1.1 to 2.1) | 2.4 (1.8 to 3) | 2.3 (1.5 to 3.3) |
| C.5.3 | Meninges Spinal Cord, Cranial Nerves, and Other parts of Central | 0 (0 to 0.1) | 0.2 (0.1 to 0.3) | 0.7 (0.4 to 1.1) | 1.4 (0.5 to 2.5) |
| C.5.4 | Nervous System | 0 (0 to 0) | 0.2 (0.1 to 0.2) | 1.3 (0.9 to 1.6) | 3.4 (2.2 to 4.8) |
| C.6 | **Female** **genital** **organs** | **21.6** **(10.9** **to** **32.5)** | **47.3** **(36** **to** **58.7)** | **63.9** **(60.4** **to** **67.3)** | **67.7** **(55** **to** **80.4)** |
| C.6.1 | Cervical cancer | 9.9 (4.9 to 15.3) | 16.3 (11.9 to 21) | 14.1 (11.9 to 16.4) | 10.8 (8 to 14) |
| C.6.2 | Corpus Uteri | 4 (1.9 to 6.3) | 11.9 (8.6 to 15.5) | 20.3 (17.5 to 23.3) | 23.9 (18.2 to 30.1) |
| C.6.3 | Other and Unspecified Female Genital Organs | 0.2 (0 to 0.7) | 0.5 (0.1 to 1) | 0.7 (0.2 to 1.2) | 0.7 (0.1 to 1.5) |
| C.6.4 | Ovarian cancer | 4.7 (2.3 to 7.4) | 12.4 (9.1 to 16.1) | 19 (16.5 to 21.6) | 20.6 (15.8 to 25.8) |
| C.6.5 | Placenta | 0.1 (0 to 0.4) | 0.1 (0 to 0.2) | 0.1 (0 to 0.1) | 0 (0 to 0.1) |
| C.6.6 | Uterine cancer | 0.7 (0.2 to 1.3) | 2.5 (1.4 to 3.7) | 5.4 (3.7 to 7.1) | 7.4 (4.4 to 10.7) |
| C.6.7 | Vagina | 0.4 (0.1 to 0.9) | 1.1 (0.5 to 1.8) | 1.8 (1 to 2.7) | 2 (0.8 to 3.4) |
| C.6.8 | Vulva | 1.6 (0.3 to 3.3) | 2.5 (1 to 4.3) | 2.5 (1.2 to 3.8) | 2.1 (0.8 to 3.7) |
| C.7 | **Leukemia** | **4.6** **(2.5** **to** **6.7)** | **13.9** **(10.9** **to** **16.9)** | **24.9** **(23.6** **to** **26.2)** | **31.1** **(26** **to** **36.2)** |
| C.8 | **Lip,** **oral** **cavity** **and** **pharynx** | **15.9** **(5.7** **to** **26.2)** | **19.5** **(13.2** **to** **25.7)** | **15.4** **(14.1** **to** **16.6)** | **11.9** **(8.9** **to** **15)** |
| C.8.1  C.8.2 | Base of Tongue Malignant neoplasm of other and ill‐defined sites in the lip, oral cavity and pharynx | 1.3 (0.1 to 3.2)  0.3 (0 to 0.7) | 0.9 (0.3 to 1.7)  0.5 (0.1 to 0.9) | 0.4 (0.2 to 0.7)  0.4 (0.2 to 0.7) | 0.2 (0.1 to 0.5)  0.3 (0.1 to 0.6) |
| C.8.3 | Floor of Mouth | 0.3 (0 to 0.8) | 0.4 (0.1 to 0.8) | 0.3 (0.1 to 0.6) | 0.2 (0 to 0.5) |
| C.8.4 | Gum | 0.4 (0 to 1.1) | 0.7 (0.2 to 1.2) | 0.7 (0.3 to 1) | 0.6 (0.2 to 1.1) |
| C.8.5 | Hypopharynx | 0.7 (0.1 to 1.6) | 1.2 (0.5 to 1.9) | 0.9 (0.5 to 1.4) | 0.7 (0.3 to 1.1) |
| C.8.6 | Lip | 5 (1.6 to 9.2) | 3 (1.7 to 4.5) | 0.7 (0.4 to 1) | 0.2 (0.1 to 0.4) |
| C.8.7 | Nasopharynx cancer | 1 (0.3 to 1.9) | 1.6 (0.9 to 2.3) | 1.3 (0.9 to 1.6) | 0.9 (0.5 to 1.3) |
| C.8.8 | Oropharynx | 0.1 (0 to 0.2) | 0.1 (0 to 0.3) | 0.1 (0 to 0.2) | 0.1 (0 to 0.2) |
| C.8.9 | Other and Unspecified Parts of Mouth | 2.8 (0.8 to 5.3) | 3.4 (2 to 5) | 2.1 (1.5 to 2.8) | 1.3 (0.8 to 2) |
| C.8.10 | Other and Unspecified Parts of Tongue | 1.9 (0.6 to 3.6) | 4.3 (2.6 to 6.1) | 4.7 (3.8 to 5.7) | 4.1 (2.8 to 5.5) |
| C.8.11 | Other and Unspecified major Salivary Glands | 0.3 (0 to 0.8) | 0.6 (0.3 to 1.1) | 0.7 (0.4 to 1) | 0.6 (0.3 to 1) |
| C.8.12 | Palate | 0.3 (0 to 0.8) | 0.5 (0.2 to 0.9) | 0.5 (0.2 to 0.8) | 0.4 (0.2 to 0.7) |
| C.8.13 | Parotid Gland | 0.5 (0.1 to 1.1) | 1.3 (0.7 to 1.9) | 1.5 (1 to 2) | 1.3 (0.8 to 2) |
| C.8.14 | Pyriform Sinus | 0.3 (0 to 0.7) | 0.3 (0.1 to 0.7) | 0.2 (0.1 to 0.4) | 0.1 (0 to 0.3) |
| C.8.15 | Tonsil | 0.5 (0.1 to 1.1) | 0.7 (0.3 to 1.2) | 0.7 (0.4 to 1) | 0.6 (0.3 to 1) |
| C.9 | **Lymphoma** | **4.3** **(1.6** **to** **6.9)** | **6.4** **(4.5** **to** **8.3)** | **5.8** **(5.3** **to** **6.3)** | **4.8** **(3.7** **to** **6)** |
| C.11 | **Other** **and** **Ill‐defined** **Sites** | **6.5** **(0** **to** **13.7)** | **6.3** **(2.7** **to** **9.8)** | **3.6** **(3.1** **to** **4.1)** | **2.3** **(1.2** **to** **3.3)** |
| C.12 | **Peripheral** **Nerves** **and** **Autonomic** **Nervous** **System** | **0** **(0** **to** **0)** | **0** **(0** **to** **0)** | **0.1** **(0** **to** **0.2)** | **1.7** **(0** **to** **4.7)** |
| C.13 | **Respiratory** **system** **and** **Intrathoracic** **organs** | **10.4** **(6.3** **to** **14.6)** | **25.8** **(20.8** **to** **30.8)** | **39.9** **(37.9** **to** **41.9)** | **46** **(39.2** **to** **52.8)** |
| C.13.1 | Accessory Sinuses | 0.4 (0.1 to 0.8) | 0.8 (0.4 to 1.2) | 0.9 (0.5 to 1.2) | 0.8 (0.4 to 1.2) |
| C.13.2 | Heart, Mediastinum, and Pleura | 0.3 (0.1 to 0.4) | 0.9 (0.6 to 1.2) | 1.7 (1.2 to 2.2) | 2.2 (1.3 to 3) |
| C.13.3 | Larynx cancer | 2.8 (1.6 to 4.2) | 5 (3.8 to 6.3) | 4.9 (4.1 to 5.7) | 4.3 (3.3 to 5.4) |
| C.13.4 | Nasal cavity and Middle Ear Other and Ill defined Sights within respiratory system and Intrathoracic | 1.2 (0.4 to 2.2) | 1.3 (0.7 to 2) | 0.8 (0.5 to 1.1) | 0.5 (0.2 to 0.8) |
| C.13.5 | Organs | 0 (0 to 0) | 0 (0 to 0.1) | 0 (0 to 0.1) | 0 (0 to 0.2) |
| C.13.6 | Thymus | 0 (0 to 0.1) | 0.1 (0 to 0.2) | 0.1 (0 to 0.3) | 0.2 (0 to 0.3) |
| C.13.7 | Trachea | 0.2 (0 to 0.4) | 0.3 (0 to 0.6) | 0.4 (0.1 to 0.7) | 0.4 (0 to 0.8) |
| C.13.8 | Bronchus, and lung cancer | 5.5 (3.3 to 7.9) | 17.4 (14 to 21) | 31.1 (29.2 to 33) | 37.6 (31.9 to 43.4) |
| C.14 | **Retroperitonem** **and** **Peritonem** | **223.4** **(77.4** **to** **370)** | **25.9** **(0** **to** **53.1)** | **0.5** **(0.4** **to** **0.7)** | **0** **(0** **to** **0.1)** |
| C.15 | **Skin** **cancer** | **105.9** **(83.9** **to** **127.9)** | **148.7** **(131.5** **to** **165.8)** | **122.4** **(118.5** **to** **126.3)** | **94.8** **(84.9** **to** **104.6)** |
| C.16 | **Thyroid** **and** **other** **endocrine** **glands** | **2.4** **(0.9** **to** **4)** | **7.5** **(5.2** **to** **9.8)** | **14.3** **(13.3** **to** **15.4)** | **18.6** **(14.2** **to** **23)** |
| C.16.1 | Adrenal Gland | 0 (0 to 0) | 0 (0 to 0.1) | 0.5 (0.2 to 0.8) | 1.8 (0.6 to 3.2) |
| C.16.2 | Other Endocrine Glands and Related Structures | 0 (0 to 0) | 0.1 (0 to 0.1) | 0.2 (0 to 0.3) | 0.3 (0 to 0.7) |
| C.16.3 | Thyroid cancer | 2.4 (0.9 to 4) | 7.4 (5.1 to 9.6) | 13.6 (12.5 to 14.7) | 16.5 (12.5 to 20.7) |
| C.17 | **Urinary** **tract** | **25.9** **(17** **to** **35.1)** | **41.7** **(34.5** **to** **49)** | **41.5** **(39.5** **to** **43.4)** | **36.8** **(31.7** **to** **41.9)** |
| C.17.1 | Bladder cancer | 23.6 (15.3 to 31.8) | 34.9 (28.8 to 41.1) | 29.8 (28 to 31.7) | 22.9 (19.5 to 26.6) |
| C.17.2 | Kidney and other urinary organ cancers | 1.8 (1.1 to 2.7) | 5.8 (4.5 to 7.2) | 10.4 (9.1 to 11.6) | 12.4 (10.2 to 14.8) |
| C.17.3 | Other and Unspecified Urinary Organs | 0.2 (0 to 0.4) | 0.2 (0.1 to 0.4) | 0.2 (0.1 to 0.4) | 0.2 (0.1 to 0.3) |
| C.17.4 | Renal Pelvis | 0.2 (0 to 0.4) | 0.5 (0.2 to 0.7) | 0.7 (0.3 to 1) | 0.8 (0.3 to 1.3) |
| C.17.5 | Ureter | 0.1 (0 to 0.3) | 0.3 (0.1 to 0.6) | 0.4 (0.2 to 0.7) | 0.5 (0.1 to 0.9) |
| C.18 | **Unknown** **Primary** **Sites** | **1.6** **(0.9** **to** **2.3)** | **12.4** **(9.7** **to** **15)** | **57.9** **(55.1** **to** **60.6)** | **124.1** **(106.4** **to** **141.8)** |
